# Analysis of Hydrodynamic Mechanism on Particles Focusing in Micro-Channel Flows

**DOI:** 10.3390/mi8070197

**Published:** 2017-06-22

**Authors:** Qikun Wang, Dan Yuan, Weihua Li

**Affiliations:** 1 School of Energy and Power Engineering, University of Shanghai for Science and Technology, Shanghai 200093, China; 2 School of Mechanical, Materials and Mechatronic Engineering, University of Wollongong, Wollongong NSW 2522, Australia; dy983@uowmail.edu.au

**Keywords:** inertial lift force, inertial focusing of particles, laminar channel flow, numerical investigation

## Abstract

In this paper, the hydrodynamic mechanism of moving particles in laminar micro-channel flows was numerically investigated. A hydrodynamic criterion was proposed to determine whether particles in channel flows can form a focusing pattern or not. A simple formula was derived to demonstrate how the focusing position varies with Reynolds number and particle size. Based on this proposed criterion, a possible hydrodynamic mechanism was discussed as to why the particles would not be focused if their sizes were too small or the channel Reynolds number was too low. The *Re*-*λ* curve (*Re*, *λ* respectively represents the channel-based Reynolds number and the particle’s diameter scaled by the channel) was obtained using the data fitting with a least square method so as to obtain a parameter range of the focusing pattern. In addition, the importance of the particle rotation to the numerical modeling for the focusing of particles was discussed in view of the hydrodynamics. This research is expected to deepen the understanding of the particle transport phenomena in bounded flow, either in micro or macro fluidic scope.

## 1. Introduction

When particles are dispersed in a laminar channel flow, they can form an annulus with a certain radius after some migration distance under proper circumstances [1]. This phenomenon shows that there are additional transverse forces exerted on the particles apart from the drag force in the flow direction, thus resulting in particles lateral migration and focusing at certain transverse positions. Since the transverse force comes from the inertia of the channel flow, it is usually regarded as the inertial lift force and the aggregative motion of the particles is called the inertial focusing of particles [2,3]. It is essentially a spontaneous hydrodynamic behavior of moving particles suspended in a laminar channel flow.

With the rapid progress in microfluidic technologies, many researchers have been attracted to study this phenomenon. This is due to its potential applications of the particle–fluid separation in microfluidic chips. Generally, this separation technique has several advantages: low energy consumption, large throughput, and high separation efficiency. Therefore, it can be used in extensive practical engineering fields, such as water purification in mechanical engineering and segregation of particles, colloids, and cells from liquid hosts in chemical and biomedical engineering, etc. [4,5,6].

However, most of relevant research on this topic has focused on the structure design and the corresponding operating parameters matching for the separation apparatus by experiments. As the determining factor on particle focusing, the inertial lift force itself has not been further studied and widely discussed yet, which is partly because of the extreme difficulty to directly measure the lift force on the moving particles inside a micro-channel flow. Instead of experimental research, there are commonly two methods to determine the magnitude of the inertial lift force. One is the approximate theoretic solution to the Navier–Stokes equations by ‘matched asymptotic expansions’ and the other is the numerical solution to the flow field by CFD (Computational Fluid Dynamics) technique.

The classical theoretic solutions to the inertial lift force were obtained using the approach of ‘matched asymptotic expansions’ to deal with Navier–Stokes equations. It was summarized that the inertial lift force *F_L_* on a spherical particle of diameter *a* in a channel of dimension *H* could be scaled as $F_{L}\propto \rho V^{2}\;a^{4}/H^{2}$, where *ρ* denotes the density of fluid and *V* is the average velocity of the channel flow [2,3,4]. However, this expression for the inertial lift force was derived on the basis of such an assumption that the size of every particle should be far smaller than that of the channel (*a* << *H*) so that the influence of particles on the flow field could be completely ignored. Therefore, this expression cannot be adopted to describe hydrodynamic behaviors of particles with finite size.

In contrast, the numerical method by CFD technique can easily break through the inherent limitation of the approximate theoretic method and has been proven to be an effective way to investigate the inertial lift force on the particles with finite size [7,8,9,10,11,12,13]. With respect to the inertial lift force, it was summarized [2,3,4,11] that the inertial lift force on a particle of finite size has two kinds of expressions, $F_{L}\propto \rho V^{2}\;a^{3}/H$ near the centerline of the channel and $F_{L}\propto \rho V^{2}\;a^{6}/H^{4}$ near its sidewall. These expressions reveal the importance of the particle size to the inertial lift force, though they are only suitable for a certain size range of particles (λ=0.22−0.38).

In many previous investigations [2,3,4,5,6], it was pointed out that the particles would not be focused if their sizes were too small or Reynolds number based on the channel size was too low. It makes sense that the hydrodynamic characteristics of the inertial lift force on particles are different in these cases. Moreover, some researchers [2,3,4] believed that the inertial lift force included mainly two components, the shear-induced lift force arising from the slope of the velocity profile of fluid and the wall-induced one from the hydrodynamic interaction between the channel wall and particles, but the effect from the particle rotation would be considered to be negligible.

This paper numerically studies the hydrodynamic mechanism of small particles of various diameters in specific low-Reynolds-number micro-channel flows. A hydrodynamic criterion was proposed to help determine whether particles in micro-channel flows can form a focusing pattern or not. An expression to demonstrate how the focusing positions vary with Reynolds number and particle size was firstly derived using the data fitting with a least square method. Based on this proposed hydrodynamic criterion, we present the mechanic explanations on why smaller particles or lower Reynolds number of the channel flow could not form a focusing pattern in such channel flows. The *Re*-*λ* curve (*Re*, *λ* respectively represents the channel-based Reynolds number and the particle’s diameter scaled by the channel) was obtained using the data fitting with a least square method so as to obtain a parameter range of the focusing pattern. Furthermore, the effects of the particle rotation on the inertial lift force were first discussed in this paper and it was proposed that the lift component induced by particle rotation has an obvious influence on the transverse focusing position of the particles, which cannot be neglected no matter for the analysis of this physical phenomenon or for its numerical modeling. This research can help deepen the understanding of the particle transport phenomena in the bounded flow in either a micro or macro scope.

## 2. Statement of the Problem and the Numerical Approach

**Figure 1:**
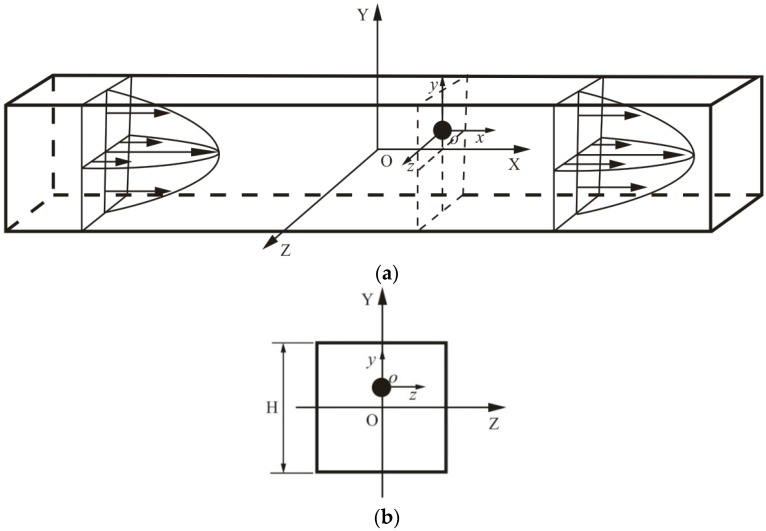
A sketch of a spherical particle in a channel flow. (**a**) a side view and (**b**) a left view.

It is assumed that a spherical particle with the diameter *a* is released in a laminar micro-channel flow driven by pressure. The density and viscosity of the fluid are *ρ* and *μ*, respectively. The channel is square in shape with a width *H*. An absolute frame (X, Y, Z) is fixed at the channel center. The sketch of a spherical migrating particle in a channel flow is plotted in Figure 1. Here, the attention is focused on some dynamic equilibria for a particle at an arbitrary transverse position of the channel, where the particle will translate along the flow direction at a constant translational velocity *U_p_* and rotate at a constant angular velocity *ω_p_*. In this situation, the inertial lift force is just a transverse component of the hydrodynamic force exerted on the particle by the surrounding fluid.

Under the absolute frame, the flow field inside the channel is unsteady with time-dependent moving boundary resulting from the movement of the particle, which is extremely difficult and time consuming for numerical computation. For computational convenience, a relative reference frame (x, y, z) is introduced, which is attached to the center of the particle and translates at the same velocity as the particle (*U_p_*) [9,10,11,12,13]. In this relative frame, the particle only rotates at the origin of the frame without translational motion, while the sidewalls of the channel move at *U_p_*, so that the flow field becomes a steady and time-independent fixed boundary if the channel is infinitely long. Furthermore, since this relative frame is essentially an inertial one, common CFD code can easily deal with the flow calculation.

In order to obtain the distribution of the inertial lift force on the particle along the *Y* coordinate, a set of standard commercially available CFD code, ‘Fluent’, was used to solve the three-dimensional steady incompressible Navier–Stokes equations. The FVM (Finite Volume Method) and SIMPLE (Semi-Implicit Method for Pressure Linked Equation) algorithm were adopted to discretize and solve the Navier–Stokes equations for the incompressible flow, where the convection terms and the viscous terms were respectively discretized by QUICK (Quadratic Upstream Interpolation for Convective Kinematics) scheme and Central scheme [14]. The computational domain was constituted such that the length of the channel was chosen as 20 times as long as the channel width (20*H*). The unstructured tetrahedral mesh was generated for the whole computational domain, and the total number of the grids was about 500,000.

To simplify the analysis, the particle was considered to translate and rotate in the symmetric plane XOY, and, it was just released in the middle section of the channel so as to reduce numerical errors induced by the inlet and outlet of the channel. A uniform-flow velocity relative to the relative frame and the ambient pressure were respectively specified as the inlet and outlet boundary conditions. The no-slip conditions relative to the relative frame were applied to both the sidewalls of the channel and the surface of the particle.

As for the computational procedure, an initial angular velocity *ω_p_* of the particle and an initial translational velocity *U_p_* of the sidewalls were set in advance as the boundary conditions on the particle surface and on the sidewalls of the channel, respectively. Then, the angular velocity *ω_p_* and the translational velocity *U_p_* of the sidewalls were iteratively updated by ‘trial and error’ according to the numerical results, until the hydrodynamic force exerted on the particle in the flow direction and the hydrodynamic torque all approach zero within the limit in numerical precision.

After the flow field was numerically simulated according to the above procedure, the stress tensor of the fluid on the particle surface could be also obtained and thus the hydrodynamic force on the particle could be obtained by Equation (1). Its component in the y direction could be easily calculated by Equation (2), which was just the inertial lift force to be further studied in this paper. In this way, the inertial lift forces on the particle at various transverse positions were numerically obtained.

(1) $$F={∯}_{\Sigma }n\cdot PdS$$

(2) $$F_{L}=F\cdot j$$

where in Equations (1) and (2),

**F** indicates the hydrodynamic force;
*F_L_* indicates the inertial lift force on the particle.
**P** indicates the fluid’s stress tensor;
Σ indicates the surface of the particle;
**n** indicates the unit vector of *dS*;
**j** indicates the unit vector of *y* axis.

To simplify the following discussions, some important coefficients were defined below.

Lift force coefficient, *C_FL_*,

(3) $$C_{FL}=\frac{F_{L}H^{2}}{\rho V^{2}\;a^{4}},$$

Dimensionless diameter of the particle, *λ*,

(4) $$\lambda =\frac{a}{H},$$

Dimensionless transverse coordinate, *y^+^*,

(5) $$y^{+}=\frac{Y}{h},$$

where *h* = *H*/2.

Channel-based Reynolds number, *Re*,

(6) $$Re=\frac{VH}{\upsilon }$$

where *υ* is the kinematic viscosity of the fluid.

## 3. Results and Discussion

### 3.1. Effects of Re and λ on the Particle Focusing

**Figure 2:**
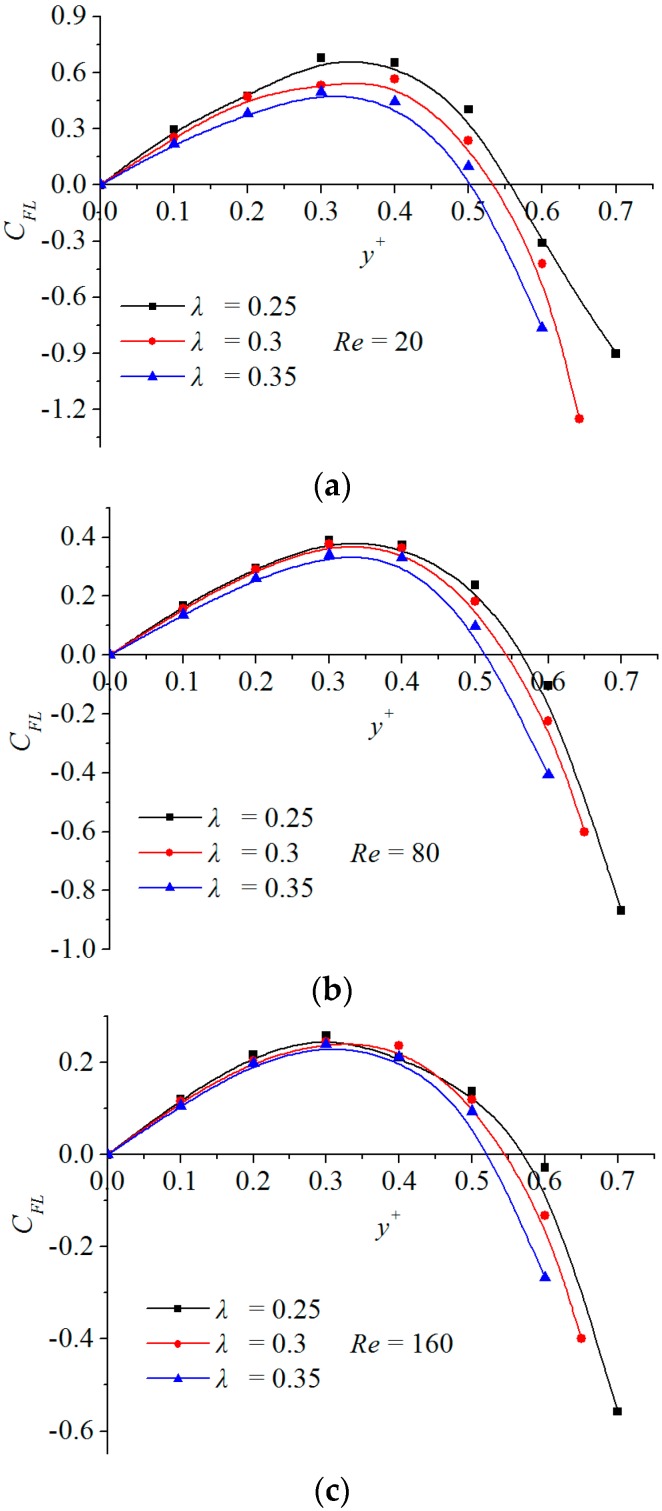
Transverse distributions of the inertial lift force coefficients of particles with different diameters against *y^+^* at various Reynolds numbers. (**a**) *Re* = 20; (**b**) *Re* = 80; and (**c**) *Re* = 160.

The transverse migration of a particle and its final dynamic equilibrium positions are determined by the inertial lift force on it. Figure 2 shows curves of inertial lift force coefficients of particles with different diameters against *y^+^* at various Reynolds numbers (*Re* = 20, 80, 160). Note from this figure that, for the given Reynolds number and particle diameter, the lift force coefficient always gradually increases firstly with *y^+^*, reaching its maximum as $y^{+}\approx 0.35$, and then it decreases monotonously until the particle nearly touches the sidewall. The inertial lift force is positive within a transverse interval $\left(0<y^{+}<0.5\right)$, indicating that it pushes the particle towards the sidewall, whereas it becomes negative and pushes the particle towards the channel center as *y^+^* is greater than a certain value.

There are two zero-inertial-lift force points along the transverse coordinate for each case, one is exactly located at the center of the channel, $y_{01}^{+}=0$ and the other is within a small interval, $y_{02}^{+}\in (0.5,0.6)$. $y_{01}^{+}$ and $y_{02}^{+}$ correspond to two zero-hydrodynamic-load positions for a spherical particle in a cross section. However, in the natural phenomena, only $y_{02}^{+}$ is the inertial focusing point. The reason is that in the vicinity of $y_{01}^{+}$, all inertial lift forces are positive pointing to the sidewall due to the symmetry of the flow. When a tiny disturbance makes the migrating particle deviate from the equilibrium point, the positive inertial lift force compels it to migrate laterally farther from its transverse position. Therefore, $y_{01}^{+}$ is an unstable dynamic equilibrium point. The situation is completely different at $y_{02}^{+}$. In its neighborhood, the inertial lift force always points at the $y_{02}^{+}$ position. When a disturbance pushes the migrating particle away from its transverse position, the inertial lift force would drive it back to its original equilibrium position. Therefore, $y_{02}^{+}$ is a stable dynamic equilibrium point, that is, a unique inertial focusing point.

Hence, not all the transverse positions with zero inertial lift force are physically stable inertial focusing positions. A physically stable focusing position must be such a transverse position which the inertial lift force in the vicinity always points to. According to the above analysis, a hydrodynamic criterion can be proposed here to determine whether the zero-inertial-lift force point $y_{0}^{+}$ obtained by the numerical method in this paper is a stable focusing position, which is mathematically expressed as

(7a) $$C_{FL}(y_{0}^{+})=0\;\mathrm{and}\;{C_{FL}}^{\prime }(y_{0}^{+})<0$$

and its dimensional form of Equation (7a) is followed as

(7b) $$F_{L}(y_{0})=0\;\mathrm{and}\;{F_{L}}^{\prime }(y_{0})<0$$

where the variable with a prime denotes its derivative with respective to transverse direction and *y_0_* denotes the dimensional coordinate Y of the zero-inertial-lift position. If a zero-inertial-lift force point satisfies Equation (7), it is a stable focusing position.

Based on the numerical approach of Section 2 and the above hydrodynamic criterion of Equation (7), the focusing positions can be numerically determined. In order to validate this method, Table 1 presents the comparisons between the numerical focusing positions by this method and the corresponding experimental results in [15]. From Table 1, it appears that the above method can obtain the reasonable focusing positions of the particle at different conditions with a relative error far below 10%, which indicates the feasibility and precision of this method for qualitative investigations.

It is also observed from Figure 2 that the focusing position varies with Reynolds number and particle size. This position would come close to the centerline of the channel as the particle size enlarges or approach the sidewall as Reynolds number increases. These two features agree well with experimental results [2,3,4,15]. Based on large amount of the calculated data obtained by the above numerical method, a simple formula can be derived using the data fitting with a least square method

(8) $$y_{0}^{+}=0.34\frac{Re{\text{​}}^{\text{​}0.0164}}{\lambda {\text{​}}^{0.22}}.$$

The formula is valid as λ∈[0.2,0.4] and Re∈[10,160], which can be used to predict behaviors of the inertial focusing of spherical particles within a corresponding parameter range of particle size and Reynolds number.

### 3.2. Features of Smaller Particles at Lower Re

It has been revealed that if the particle sizes are too small relative to the scale of a channel or Reynolds number based on the channel scale is too low, the migrating particles would not be focused [2,3,4,5,6]. However, it has not yet been explained why the migrating behaviors of smaller particles are considerably different from those of larger ones in a low-Reynolds-number channel flow, and what the proper range is of parameters *Re* and *λ* for particle focusing.

**Figure 3:**
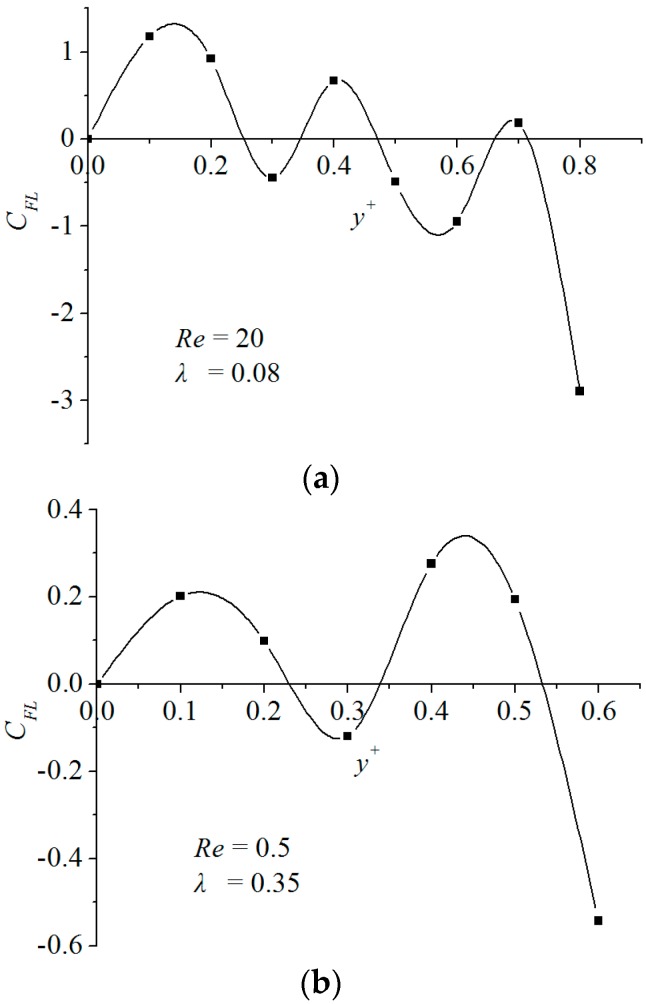
Transverse distributions of the inertial lift force: (**a**) is for smaller particle and (**b**) is for lower *Re.*

Figure 3 presents the transverse distributions of the inertial lift force for a smaller particle or a lower Reynolds number case. It is seen from Figure 3a that the inertial lift force curve of a smaller particle size appears in a fluctuation pattern along the transverse direction and has many null points. For all these zero points, some satisfy the hydrodynamic criterion of Equation (7) while the others do not. This means that there coexist many stable and unstable focusing points within a transverse interval. Therefore, particles with smaller size do not always migrate at a stable focusing position, which makes the focusing of particles unobservable. Similar behaviors can be observed in Figure 3b for a lower Reynolds number case. Figure 3b shows that when *Re* decreases to a lower level, for example, Re=0.5, multiple inertial-lift force null points would occur. According to Equation (7), a single focusing does not appear for such a particle size, although λ=0.35.

**Figure 4:**
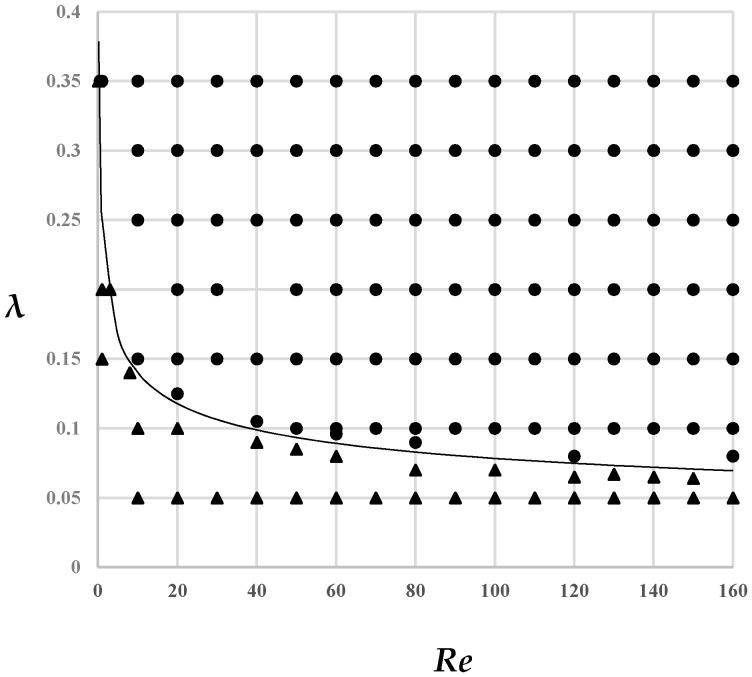
Inertial focal points on the parameter plane of *Re* and *λ*, ● for focusing points and ▲ for unfocusing ones.

Since parameters *Re* and *λ* affect the phenomenon of the particles focusing, the proper range of *Re* and *λ* for particle focusing were investigated. Figure 4 numerically presents focusing points and unfocusing points on the parameter plane of *Re* and *λ*.

Note from this figure that the *Re*-*λ* parameter plane is divided by a boundary curve into two parts, the focusing region and unfocusing one. Since this boundary curve in Figure 4 presents a hyperbolic-like style, the equation for this curve is assumed as *Reλ^n^* = *C*. The two unknown constants *n* and *C* can be determined by data fitting with a least square method, and the final result is [16].

(9) $$Re\cdot \lambda^{4}=0.004315$$

Equation (9) confines a parameter region of the focusing pattern of migrating smaller particles in a low-Reynolds-number channel flow.

In the view of practical application, the left part of Equation (9) can be regarded as a new definition for the particle Reynolds number *Re_p_* shown as Equation (10) and the right part of Equation (9) can be regarded as the corresponding critical value for the particle Reynolds number *Re_p_cr* described in Equation (11). Equation (12) can be regarded as the criterion to judge whether the focusing of particles can occur or not.

(10) $$Re_{p}=Re\cdot \lambda^{4}$$

(11) $$Re_{p}cr=0.004315$$

(12) $$Re_{p}\geq Re_{p}cr$$

When the particle Reynolds number exceeds its critical value, particles can be stably focused otherwise no focusing of particle occurs. This is an empirical but useful way to predetermine whether or not a certain scale of particle can be focused inside a channel flow with certain Reynolds Number.

### 3.3. Rotational Effect of Particles

The inertial lift force on a migrating particle in a bounded flow is usually considered to be composed of three parts [2,3]: the shear-induced lift force, which results from the velocity gradient of the flow and points to its opposite direction (Saffman lift force); the wall-induced lift force, which is a transverse propulsive force generated by the sidewall; and rotation-induced lift force, which is exerted on the particle by the fluid due to its translation associated with rotation (Magnus lift force). However, it was believed that the rotational effect was not significant in the low-Reynolds-number channel flow [2,3], and thus its influence on the focusing of particles was neglected.

Induced by the velocity gradient of the flow field inside a channel, the particle rotation may always spontaneously occur accompanying with the translational motion of the particles. It is too difficult to control a moving particle from rotating in experiments. However, it can be easily fulfilled in numerical calculation, since the angular velocity *ω_p_* of the rotation of the particle can be easily set to zero during the numerical calculation. In this way, the inertial lift force can be obtained without the consideration of particle rotation. By comparison with the corresponding results considering the particle rotation in Section 3.1, the particle rotation effect on the inertial lift force may be revealed and evaluated.

**Figure 5:**
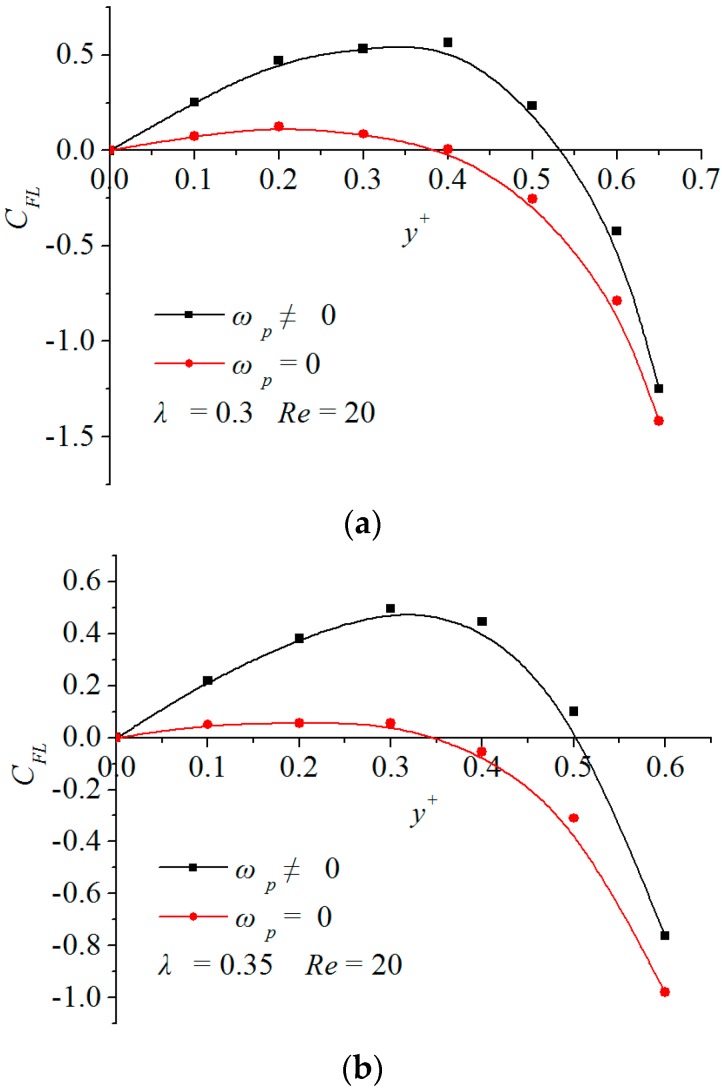
Rotational effect of particles on the inertial lift force: (**a**) is for *λ* = 0.3 and (**b**) is for *λ* = 0.35 at *Re* = 20.

Figure 5 shows the particle rotation effect on the inertial lift forces. The case of $\omega_{p}\neq 0$ corresponds to dynamic equilibriums under the consideration of the particle rotation, whereas that of $\omega_{p}=0$ only corresponds to dynamic equilibriums under no consideration of the particle rotation.

The difference between the two cases reveals the contribution of the particle rotation on the inertial lift force. Note from the figure that the two inertial-lift force curves are quite different from each other. This implies that the rotation effect of particles has a significant influence on their inertial lift forces. When a particle migrates without rotation, the inertial lift force attenuates and its zero-inertial-lift force point $y_{0}^{+}$ comes closer to the centerline of the channel, that is, the rotation-induced lift force always points to the sidewall of the channel and makes the stable focusing position deviate from the centerline of the channel.

**Figure 6:**
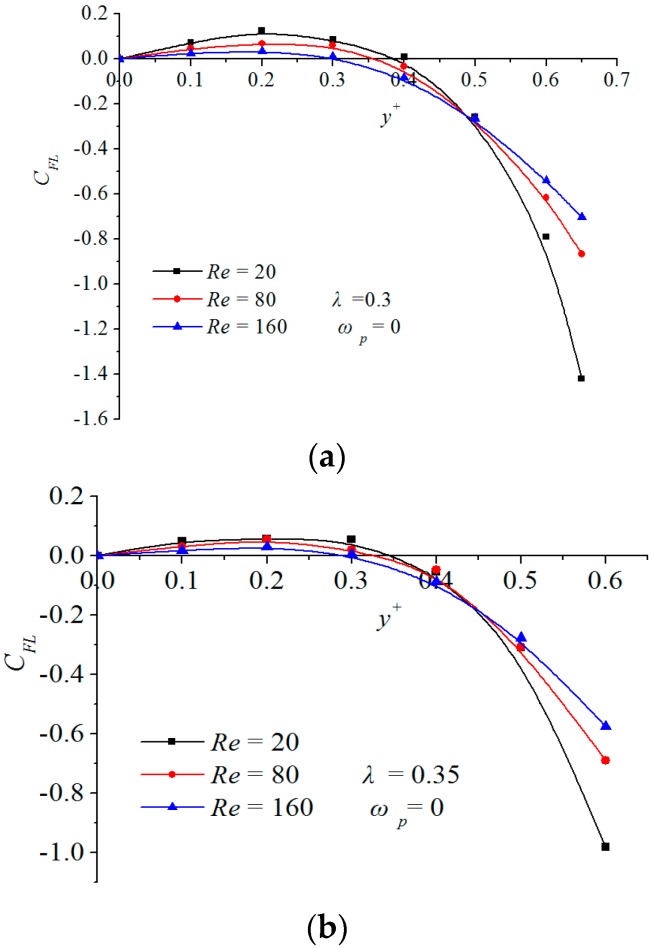
Transverse distributions of the inertial lift force on a migrating particle without rotation at various Reynolds numbers. (**a**) is for *λ* = 0.3 and (**b**) is for *λ* = 0.35 at different *Re* of 20, 80, 160.

Influence of the Reynolds number on the inertial lift force on a migrating particle without rotation is plotted in Figure 6. It is observed from Figure 6 that when a particle migrates without rotation, the zero-inertial-lift force point $y_{0}^{+}$ comes closer to the centerline of the channel with the increment of *Re*. This is inverse to the case with rotational effect shown in Figure 2. Therefore, if the rotation-induced lift force is ignored in the numerical modeling, the physical phenomenon cannot be correctly described at all. In summary, the lift component induced by the particle rotation has an obvious influence on the transverse focusing position of the particles, which cannot be neglected no matter the analysis of physical phenomenon or the numerical modeling.

## 4. Conclusions

The paper numerically investigates hydrodynamic behaviors of small particles in laminar channel flows. A hydrodynamic criterion was proposed to help determine whether particles in channel flows can form an inertial-focusing pattern or not. In view of the numerical computation combined with the corresponding experimental data, a simple formula was derived using data fitting with a least square method to demonstrate how inertial-focusing position varies with Reynolds number and particle size. The formula is valid when λ∈[0.2,0.4] and Re∈[10,160]. Moreover, based on this proposed hydrodynamic criterion, the mechanic explanations were presented as to why the particles would not be focused if their sizes were too small or the channel Reynolds number was too low. Then, a *Re*-*λ* curve expression was obtained using data fitting with a least square method, which presents a boundary curve to confine a parameter range of the inertial-focusing pattern. Finally, the influence of particle rotation on its focusing position was discussed at various Reynolds numbers to stress the importance of the rotation-induced lift force to the focusing of particles.

## Figures and Tables

**Table 1:** Validation of the focusing position.

Re/*a*^+^	$y_{0}^{+}$ (Experimental)	$y_{0}^{+}$ (Numerical)	**Error (%)**
10/0.25	0.590	0.550	6.78
10/0.35	0.540	0.510	5.56
40/0.35	0.578	0.540	6.57
210/0.11	0.780	0.827	6.03
360/0.095	0.797	0.831	4.27
